# An exploration of medical student attitudes towards disclosure of mental illness

**DOI:** 10.1080/10872981.2020.1727713

**Published:** 2020-02-13

**Authors:** Ian Fletcher, Michael Castle, Aaron Scarpa, Orrin Myers, Elizabeth Lawrence

**Affiliations:** a School of Medicine, University of New Mexico, Albuquerque, USA; b Department of Family and Community Medicine, University of New Mexico School of Medicine, Albuquerque, NM, USA; c Department of Internal Medicine, University of New Mexico School of Medicine, Albuquerque, NM, USA

**Keywords:** Student wellness, medical student, mental health, mental health disclosure, mental Illness, medical licensure

## Abstract

**Background**: Medical students are reluctant to access mental health services, despite having high rates of anxiety and depression. This reluctance persists through residency and into practice. Physicians and trainees who are unwell deliver lower quality patient care, behave less professionally, communicate less effectively and are at an increased risk for burnout and suicide. Little is known about whether students would disclose a mental health diagnosis on a state board medical license application.

**Objectives**: The objectives of this study were to determine whether University of New Mexico School of Medicine (UNM SOM) students would be willing to disclose a mental health diagnosis on a medical licensing application if prompted to do so, and, if not, to identify the reasons for their unwillingness to do so.

**Design**: We electronically invited all UNM SOM students enrolled in the Classes of 2019, 2020, 2021, and 2022 to participate in a confidential RedCap survey about mental health diagnoses and treatment. Four e-mail invitations and reminders were sent to students over a one-month period.

**Results**: Response rate was 50.1%. Thirty-six percent of all respondents considered themselves to have had a mental health condition prior to medical school, and 47% of all respondents perceived a decline in mental health during medical school. The majority of respondents who perceived they had a mental health diagnosis (51%) stated they would not disclose this information on a New Mexico Medical Board (NMMB) license application. Fear of stigmatization, fear of repercussions, and a belief that such disclosure was irrelevant were the top reasons for non-disclosure.

**Conclusion**: Students who perceive themselves to have mental health diagnoses are unlikely to disclose their mental health status on state medical board licensing applications when asked to do so. Addressing barriers to disclosure of mental health diagnoses is necessary for building a healthier physician workforce.

## Introduction

Medical students experience high rates of distress, burnout, anxiety, depression and suicidal ideation [1–4], and yet they are reluctant to seek mental health services [1,5,6,7–11]. Barriers to help-seeking include concerns about confidentiality, time, cost, perceived stigma, potential repercussions, and unwanted interventions [12,13].

Practicing physicians and medical residents cite many of these same reasons for their own reluctance to receive needed counseling or to take recommended medications for a mental health diagnosis [14–17]. Physicians practicing in states in which state medical licensure applications inquire about prior mental health conditions are less likely to access recommended mental health services than physicians in states in which such questions are not asked [18].

The wellbeing of the physician workforce is important for patient care. Physicians who are burned out, for example, are twice as likely to practice medicine unsafely, demonstrate unprofessional behaviors and receive lower patient satisfaction scores [19].

Little is known about whether medical students are willing to self-disclose a history of mental health diagnosis or treatment if prompted to do so on a state licensure application or other professional application. At the time our study was conceived, New Mexico was one of the 24 states with a medical board that queried about past as well as current diagnosis or treatment of a mental health condition [18]. Since approximately 38% of UNM SOM graduates eventually practice medicine in the state of New Mexico [20], we believed that data about UNM SOM students’ willingness or unwillingness to disclose mental health conditions might encourage the New Mexico Medical Board (NMMB) to modify language in its licensing application to limit inquiry regarding mental health conditions to current impairment. Such language complies with recommendations of the American Medical Association in 2018 [21] and the Federation of State Medical Boards in 2018 [22].

We therefore chose to investigate the question of whether medical students are willing to disclose mental health diagnoses on medical licensure applications and residency applications through the Electronic Residency Application System (ERAS). We chose to include residency applications although such applications do not presently inquire about past mental health diagnosis or treatment due to our students’ repeatedly expressed unwillingness to seek mental health services because they ‘wanted to match.’ We investigated the possible reasons for students’ unwillingness to disclose mental health diagnoses or treatment. We hypothesized that our students would either avoid accessing mental health support services or fail to disclose the use of those services out of fear of professional repercussions.

## Methods

### Recruitment and participants

We invited all currently enrolled UNM SOM students (Classes of 2019, 2020, 2021, 2022) to complete a brief anonymous electronic survey administered on the RedCap online platform. Student’s responses were collected and stored on the RedCap platform, and basic data summaries were performed by the program [23].

Three hundred forty-nine students were invited via an email containing a description of the study and a link to the survey in the RedCap platform (Appendix 2). Three reminder emails that differed in subject headings only were sent to the students during the month-long survey period. No incentives were given or implied in the recruiting of students, and no additional efforts were made to recruit participants. The study was approved by the University of New Mexico Health Sciences Human Research Protections Program (IRB study number: 18–157). No identifying information was used at any point in this study and, due to the voluntary nature of the survey, consent was implied when a student responded to the survey.

### Survey components

The study survey asked students whether they considered themselves to have had a mental health condition prior to matriculation, whether they perceived a change in their mental health during medical school, whether they were willing to use mental health services, and whether they would disclose a mental health diagnosis on a licensing application to the NMMB or on ERAS. Students who responded that they were undecided about disclosing or would decline to disclose were asked to identify reasons for non-disclosure. These follow up questions were adapted with written permission from a survey about disclosure of mental health issues developed by Katherine J. Gold, MD, MSW, a family physician and associate professor at the University of Michigan Departments of Family Medicine and Obstetrics & Gynecology. Dr. Gold developed the survey based on the available literature about physicians, mental health, and state licensing. She used the survey to explore female doctors’ attitudes about mental health disclosure to their medical boards. Prior to Dr. Gold’s survey, there were no validated surveys about physician or trainee attitudes about disclosure to state medical boards. The questions in our survey can be found in Appendix 1.

### Statistical analyses

Responses were summarized as frequencies, percentages, and means. Standard deviations were used for numeric variables. Associations between categorical variables were analyzed using contingency table analyses (Chi-square, Fisher exact test) and by multivariate logistic regression. Non-parametric Wilcoxon rank sum tests and Kruskal–Wallis tests were used to assess equality of medians across groups. Agreement between disclosure patterns to residency versus to licensure was tested using Bowker’s symmetry test. SAS v9.4 was used for analyses.

## Results

We had a 50.1% response rate, with 175 of the 349 invited students completing the survey. Sixty-three of the 175 respondents (36%) were first-year students, 37 respondents (21.1%) were second-year students, 45 respondents (25.7%) were third-year students, and 30 respondents (17.1%) were fourth-year students.

Of the students who responded, 36% reported that they perceived they had a mental health condition prior to medical school. Forty-seven percent of respondents noted an overall worsening of their perceived mental health throughout their medical training (*P* < .001). The odds ratios of a second-year, a third-year, or a fourth-year medical student believing that his or her mental health had worsened since entering medical school versus improved or remained the same, with first-year medical students as the referent, were 3.64 (95% CI 1.5–8.8), 8.52 (95% CI 3.54–20.49), and 7.69 (95% CI 2.91–20.37), respectively.

Students used a Likert scale of one to the ten (one being very unlikely and ten being very likely) to describe the likelihood of seeking treatment for a new or ongoing mental health condition while enrolled in medical school. Twenty-three percent were unlikely to seek access to treatment (score ≤3) and 34% were likely to seek access to treatment (score ≥8).

We assessed medical student willingness to disclose a diagnosis of a mental health condition on NMMB and ERAS applications if asked to do so. Fifty-one percent of students reported that they would not disclose their mental health information on the NMMB medical licensure application, while 21% of students said they would disclose. For ERAS, only 7% of students reported they would disclose, as opposed to 63% who would not disclose if prompted. There was no difference between the number of years in medical school and the likelihood of seeking access to treatment, the likelihood of NMMB disclosure, or the likelihood of ERAS disclosure (Table 1). The reasons for not disclosing to ERAS and the NMMB can be found in Tables 2 and 3.

**Table 1.** Student question response by year (n = 175)

	First Year (*n* = 63)		Second Year (*n* = 37)		Third Year (*n* = 45)		Fourth Year (*n* = 30)		
	N	%	N	%	N	%	N	%	P-value
Prior to attending the UNM School of Medicine would you have considered yourself to have a mental health condition. (i.e. depression, anxiety, bipolar disorder, schizophrenia, obsessive compulsive disorder, eating disorders, personality disorders, ADHD, etc.)?									
Yes	22	34.9	14	37.8	14	31.1	13	43.3	0.906
No	34	54.0	18	48.6	27	60.0	15	50.0	
Unsure	7	11.1	5	13.5	4	8.9	2	6.7	
How would you assess the state of your mental health now during your time in medical school as compared to the beginning of medical school?									
No change	37	58.7	12	32.4	8	17.8	6	20.0	<0.001
Better	13	20.6	7	18.9	6	13.3	4	13.3	
Worse	13	20.6	18	48.6	31	68.9	20	66.7	
On a scale of 1–10(1 very unlikely- 10 very likely), please reflect how likely you would be to seek access to treatment for a new or ongoing mental health condition while enrolled in medical school?									
1-3	11	17.5	11	29.7	13	28.9	4	13.3	0.286
4-7	33	52.4	15	40.5	16	35.6	12	40.0	
8-10	19	30.2	11	29.7	16	35.6	14	46.7	
If you were to have a diagnosis for a mental health condition would, you be willing to disclose the condition when applying to residency through the Electronic Residency Application Service (ERAS)?									
Yes	6	9.5	3	8.1	2	4.4	1	3.3	0.881
No	40	63.5	23	62.2	27	60.0	21	70.0	
Unsure	17	27.0	11	29.7	16	35.6	8	26.7	
If you were to have a diagnosis for a mental health condition, would you be willing to fully disclose this information to the NMMB on the application for licensure with current wording as stated: In the five [18] years prior to this application									
Yes	13	20.6	10	27.0	8	17.8	5	16.7	0.898
No	30	47.6	18	48.6	25	55.6	17	56.7	
Unsure	20	31.7	9	24.3	12	26.7	8	26.7

**Table 2.** Reasons for not disclosing to ERAS (n = 175)

Reason	*n*	%
Fear of being stigmatized	115	65.7
Fear of not matching	108	61.7
Desire to be viewed more competitively on the application	107	61.1
Fear of repercussion or judgment	100	57.1
Did not think condition was the business of ERAS	86	49.1
Opinion that it is not applicable to your performance or ability	83	47.4
Fear of being questioned in residency interviews	74	42.3
Fear of other people besides the program director(s) finding out	41	23.4
Did not want to do follow-up paperwork	40	22.9
Did not want to be placed in physician health program	39	22.3
Knowledge that your condition is currently controlled on treatment	33	18.9
Did not disclose on other applications and wanted to be consistent	15	8.6
N/A	14	8.0

**Table 3.** Reasons for not disclosing to NMMB (*n* = 175)

Reason	*n*	%
Fear of being stigmatized	87	49.7
Belief that this is not applicable to your performance or ability	81	46.3
Fear of repercussion or inconveniences (eg, extra paperwork)	80	45.7
Fear of having to do more follow-up paperwork	76	43.4
Fear of being questioned in interviews	75	42.9
Belief that your current condition did not pose a safety risk to patients	66	37.7
Knowledge that your condition is currently controlled on treatment	46	26.3
Fear that you could not get an unrestricted license	39	22.3
Fear of being placed in a physician health program	36	20.6
Fear others outside the medical board would find out	30	17.1
Belief that your current condition was not the business of medical board	25	14.3

Medical students without a history of a mental health condition were less likely to report a worsening of mental health status during medical school (Table 4, *P* = .013). Students with a history of a perceived mental health diagnosis were the least likely to disclose to the NMMB (*P* = .008). There was not a significant difference in likelihood to disclose to ERAS among students with a history of a perceived mental health condition and those without such a history (*P* = .015).

**Table 4.** Student question responses by mental health status (n = 175)

	Prior to attending the UNM School of Medicine would you have considered yourself to have a mental health condition. (i.e. depression, anxiety, bipolar disorder, schizophrenia, obsessive compulsive disorder, eating disorders, personality disorders, ADHD, etc.)							
	Yes (*n* = 63)		No (*n* = 94)		Unsure (*n* = 18)			
	*n*	%	*n*	%	*n*	%	P-value	
How would you assess the state of your mental health now during your time in medical school as compared to the beginning of medical school?								
No change	14	22.2	44	46.8	5	27.8	0.013	
Better	16	25.4	10	10.6	4	22.2		
Worse	33	52.4	40	42.6	9	50.0		
On a scale of 1–10(1 very unlikely- 10 very likely), please reflect how likely you would be to seek access to treatment for a new or ongoing mental health condition while enrolled in medical school?								
1-3	10	15.9	25	26.6	4	22.2	0.072	
4-7	23	36.5	43	45.7	10	55.6		
8-10	30	47.6	26	27.7	4	22.2		
If you were to have a diagnosis for a mental health condition would, you be willing to disclose the condition when applying to residency through the Electronic Residency Application Service (ERAS)?								
Yes	7	11.1	5	5.3	0	0.0	0.146	
No	35	55.6	66	70.2	10	55.6		
Unsure	21	33.3	23	24.5	8	44.4		
If you were to have a diagnosis for a mental health condition, would you be willing to fully disclose this information to the NMMB on the application for licensure with current wording as stated: In the five [18] years prior to this application?								
Yes	5	7.9	26	27.7	5	27.8	0.008	
No	38	60.3	47	50.0	5	27.8		
Unsure	20	31.7	21	22.3	8	44.4		

We used multivariate logistic regression to evaluate the joint effects of prior history, year, and likelihood of seeking access to treatment on probability of worsening mental health status. Only school year was associated with self-reported worsening mental health (*P* < 0.001 OR_2nd year_ = 3.70 (95% CI = 1.50 to 9.12), OR_3rd year_ = 9.32 (95% CI = 3.227 to 23.04), and OR_4th year_ = 7.84 (95% CI = 2.91 to 21.10).

## Discussion

An average of 37.8% of UNM SOM medical students perceived that they had a mental health condition prior to matriculation, a percentage that is consistent with what has previously been described in the literature [24]. Almost half of UNM SOM medical students perceive their mental health to have deteriorated as they progressed through undergraduate medical training. This finding is consistent with other reports [1,25] and has been attributed to excessive workload, pressure to succeed, fatigue, ethical conflicts, accumulating debt, and exposure to death and human suffering [3,8,26,27].

Gold *et al*. surveyed licensed physicians about reasons for nondisclosure of mental illness on state medical licensure forms and found that fear of stigmatization accounted for only 5% of the responses [15]. In our study, 65.7% of respondents cited fear of being stigmatized as a reason for nondisclosure. The most likely explanation for this difference is that earlier in medical training, students are more sensitive about how others perceive them [6,10,28,29]. Students may also be less knowledgeable about the actual repercussions and more likely to believe rumors such as, ‘If I ever take a Prozac, I can’t be a doctor.’ The differences in how medical students and physicians imagine and fear stigmatization demonstrates how future interventions need to be targeted to specific populations, i.e., to medical students, residents, or practicing physicians.

Questions about mental health history on licensure and other professional applications are presumably designed to identify physicians and trainees who pose significant patient safety concerns [30,31]. Such inquiries create a barrier, however, to current and future physicians who would seek mental health care or treatment [15,18]. Ultimately, these inquiries unwittingly create future physician health and patient safety concerns by discouraging physicians from seeking needed mental health diagnosis and treatment. Our study provides evidence that medical students are more likely than not to withhold information about mental health diagnosis on licensure and residency applications.

Fortunately for New Mexico medical students, residents, and physicians, the NMMB has modified the wording of its initial and renewal medical licensure applications. The new question about mental health diagnosis asks, ‘Do you have or have you been diagnosed with an illness or condition which impairs your judgement or affects your ongoing ability to practice medicine in a competent, ethical, and professional manner?’ [32]. Unlike the previous language in the licensing application [33], this less stigmatizing language is consistent with recommendations of the American Medical Association in 2018 [21] and the Federation of State Medical Boards in 2018 [22].

### Limitations

Our study is a single-site study, and the applicability of our findings to other institutions is unknown. Our data were collected at one point in time, and we are not able to follow cohorts prospectively due to the confidential nature of the survey. We are therefore unable to know definitively whether the higher rates of self-reported mental illness in the third-year class points to individual class characteristics or a trend seen in students progressing through medical school. We believe the latter to be true, given its consistency with other studies [26,27,34,35], the consistency of our admissions processes for the classes in question, and the improvement in perceived mental health status for fourth-year students compared with third-year students. We plan to address cohort-effects and inability to prospectively follow medical school classes in a future study described below.

Our e-mail invitation to participate in the study appears in Appendix 2. It was sent to all members of the Classes of 2019, 2020, 2021, and 2022. Our survey response rate was 50.1%, a rate consistent with that seen in other studies relating to mental health in medical professionals [3,5,17]. The students who elected to complete the survey may have had more interest in mental health issues than non-respondents, may have been more likely to have received a mental health diagnosis or treatment, or may have been less likely to have received a mental health diagnosis or treatment. We did not collect sufficient demographic data to compare medical student respondents and non-respondents, and our study is therefore limited by selection bias. The voluntary nature of survey completion, however, should reduce response bias.

The final major limitation in our study is the use of a non-validated questionnaire. Some of our questions were copied from or based on a published study, with permission from the author [15], and were based on the limited literature available on mental health, physician wellness, licensing applications, and disclosure attitudes. To our knowledge, there is no validated study that we could have used in place of the survey we created.

### Future research

The authors have completed a second year of data collection with the goal of conducting a five-year longitudinal study. This longitudinal study and its consequently larger sample of survey respondents will better enable us to analyze potential cohort effects, increase the overall sample size, and minimize selection bias. A multi–institutional study involving medical schools in states in which medical boards continue to probe mental health issues on medical licensing applications might provide needed encouragement to medical boards to reduce or eliminate stigmatizing language. Another possible area for future study is to explore how language consistent with national recommendations for inquiry into current impairment, such as the language now on the NMMB license application, affects the willingness of students, residents, and physicians to avail themselves of mental health services and disclose this history.

## Conclusion

This study suggests that medical students, much like practicing physicians and residents, are reluctant to use mental health services and to disclose a history of mental health diagnosis. Students at UNM SOM, consistent with what has been shown in larger studies of medical students, perceive a worsening in their mental health as they progress through their medical education. Decreasing the stigma associated with using mental health resources, in part by modifying the language in common professional applications to inquire only about current impairment, is a necessary step toward building a healthier physician workforce.

## Appendix 1: Survey

**Table:**

1. Please specify what year in medical school you currently are in: **a**. 1st year **b**. 2nd year **c**. 3rd year **d**. 4^th^ year 2. Prior to attending the UNM School of Medicine would you have considered yourself to have a mental health condition (i.e. depression, anxiety, bipolar disorder, schizophrenia, obsessive-compulsive disorder, eating disorders, personality disorders, ADHD, etc.)? **a**. Yes **b**. No **c**. Unsure 3. How would you assess the state of your mental health now during your time in medical school as compared to the beginning of medical school? **a**. No change **b**. Better **c**. Worse 4. On a scale of 1–10 (1 very unlikely- 10 very likely), please reflect how likely you would be to seek access to treatment for a new or ongoing mental health condition while enrolled in medical school? 5. If you were to have a diagnosis for a mental health condition, would you be willing to disclose the condition when applying to residency through the Electronic Residency Application Service (ERAS)? **a**. Yes **b**. No **c**. Unsure 5a. If you answered no or unsure to the above question please select the reasons from the following list. (Select all that apply). a. The fear of being stigmatized b. The desire to be viewed more competitively on the application c. The fear of repercussion or judgment d. The opinion that it is not applicable to your performance or ability to safely care for patients e. The knowledge that your condition is currently controlled on treatment f. The fear of being questioned in residency interviews g. The fear of not matching h. The fear of other people besides the program director(s) finding out i. Did not think condition was the business of ERAS j. Did not want to do follow-up paperwork k. Did not want to be placed in physician health program l. Did not disclose on other applications and wanted to be consistent m. N/A 6. If you were to have a diagnosis for a mental health condition, would you be willing to fully disclose this information to the NMMB on the application for licensure with current wording as stated: *‘In the five [18] years prior to this application, have you had any physical injury or disease, or mental illness or impairment, which you are currently under treatment for or could reasonably expect to affect your on-going ability to practice medicine safely and competently?’** a. Yes b. No c. Unsure 6a. If you answered no or unsure to the above question please select the reasons from the following list. (Select all that apply). a. The fear of being stigmatized b. The fear of repercussion or inconveniences (e.g., extra paperwork) c. The belief that this is not applicable to your performance or ability d. The knowledge that your condition is currently controlled on treatment e. The fear of being questioned in interviews f. The belief that your current condition did not pose a safety risk to patients g. The belief that your current condition was not the business of medical board h. The fear of having to do more follow-up paperwork i. The fear that you could not get an unrestricted license j. The fear of being placed in a physician health program k. The fear others outside the medical board would find out L. N/A *This wording was the original language in the New Mexico Board of Medical Examiners new and renewal physician licensure application.

## Appendix 2: Email Invitation to all members of the Classes of 2019, 2020, 2021, 2022

University of New Mexico School of Medicine students,

Dr. Elizabeth Lawrence, Ian Fletcher, Michael Castle, and Aaron Scarpa of the Class of 2020 are conducting a research study to assess attitudes about disclosing information about mental illness/treatment/diagnosis on both residency and state medical licensing applications. Your participation is completely voluntary, and you may choose to not participate. Your responses will be completely ANONYMOUS. No names or identification are associated with this survey.

Your participation will include completing a survey via the platform RedCap and should take less than 5 min to complete. This survey includes questions about mental illness diagnosis status, and ease with which individuals feel disclosing such information on the aforementioned applications. We understand that some individuals may experience discomfort when answering these questions, but we wish to reiterate that responses will be completely anonymous.

If you have any questions, please contact any of the individuals associated with this project.

We thank you in advance for your participation.

Ian Fletcher

Michael Castle

Aaron Scarpa

Elizabeth C. Lawrence, MD*

*Contact information for three students and Dr. Lawrence was provided in the original message sent to students.
